# Definition of the Cattle Killer Cell Ig–like Receptor Gene Family: Comparison with Aurochs and Human Counterparts

**DOI:** 10.4049/jimmunol.1401980

**Published:** 2014-11-14

**Authors:** Nicholas D. Sanderson, Paul J. Norman, Lisbeth A. Guethlein, Shirley A. Ellis, Christina Williams, Matthew Breen, Steven D. E. Park, David A. Magee, Farbod Babrzadeh, Andrew Warry, Mick Watson, Daniel G. Bradley, David E. MacHugh, Peter Parham, John A. Hammond

**Affiliations:** * The Pirbright Institute, Pirbright, Woking, Surrey GU24 0NF, United Kingdom;; † Department of Structural Biology, Stanford University, Stanford, CA 94035;; ‡ Department of Microbiology and Immunology, Stanford University, Stanford, CA 94035;; § Department of Molecular Biomedical Sciences, College of Veterinary Medicine, North Carolina State University, Raleigh, NC 27695;; ¶ Center for Comparative Medicine and Translational Research, Raleigh, NC 27539;; ‖ Lineberger Comprehensive Cancer Center, Chapel Hill, NC 27599;; # Animal Genomics Laboratory, School of Agriculture and Food Science, College of Agriculture, Food Science and Veterinary Medicine, University College Dublin, Belfield, Dublin 4, Ireland;; ** Stanford Genome Technology Center, Palo Alto, CA 94304;; †† Bioscience Information Technology Services, Biotechnology and Biological Sciences Research Council, Swindon SN2 1UH, United Kingdom;; ‡‡ The Roslin Institute and Royal (Dick) School of Veterinary Studies, University of Edinburgh, Easter Bush Campus, Midlothian EH25 9RG, United Kingdom;; §§ Smurfit Institute of Genetics, Trinity College, Dublin 2, Ireland; and; ¶¶ Conway Institute of Biomolecular and Biomedical Research, University College Dublin, Belfield, Dublin 4, Ireland

## Abstract

Under selection pressure from pathogens, variable NK cell receptors that recognize polymorphic MHC class I evolved convergently in different species of placental mammal. Unexpectedly, diversified killer cell Ig–like receptors (KIRs) are shared by simian primates, including humans, and cattle, but not by other species. Whereas much is known of human KIR genetics and genomics, knowledge of cattle KIR is limited to nine cDNA sequences. To facilitate comparison of the cattle and human *KIR* gene families, we determined the genomic location, structure, and sequence of two cattle *KIR* haplotypes and defined *KIR* sequences of aurochs, the extinct wild ancestor of domestic cattle. Larger than its human counterpart, the cattle *KIR* locus evolved through successive duplications of a block containing ancestral *KIR3DL* and *KIR3DX* genes that existed before placental mammals. Comparison of two cattle *KIR* haplotypes and aurochs *KIR* show the *KIR* are polymorphic and the gene organization and content appear conserved. Of 18 genes, 8 are functional and 10 were inactivated by point mutation. Selective inactivation of *KIR3DL* and activating receptor genes leaves a functional cohort of one inhibitory KIR3DL, one activating KIR3DX, and six inhibitory KIR3DX. Functional KIR diversity evolved from *KIR3DX* in cattle and from *KIR3DL* in simian primates. Although independently evolved, cattle and human *KIR* gene families share important function-related properties, indicating that cattle KIR are NK cell receptors for cattle MHC class I. Combinations of *KIR* and *MHC class I* are the major genetic factors associated with human disease and merit investigation in cattle.

## Introduction

Genes of the immune and reproductive systems exhibit the most variation between mammalian genomes (1, 2). This variability reflects the strong selection pressures imposed by the vital functions of immunity and reproduction. NK cells are a diverse and unique population of lymphocytes that contribute to both immunity and reproduction. In the defense against infection, particularly viral infections, NK cells are the principal lymphocytes of the innate immune response (3). They kill virus-infected cells and secrete cytokines that recruit other leukocytes to the infected tissue. NK cells also help initiate the adaptive immune response and make prominent contributions to the control and elimination of cancer (4, 5). In reproduction, NK cells cooperate with extravillous trophoblast cells in formation of the placenta, the organ that provides the growing fetus with nourishment throughout pregnancy (6).

Unlike B and T lymphocytes, NK cells do not express variable Ag receptors made from rearranging genes. Instead, NK cells express different combinations of a variety of different cell-surface receptors, many of which are encoded by genes in either the leukocyte receptor complex (*LRC*) or the NK complex (*NKC*), gene complexes on different chromosomes: for example, human chromosomes 19 and 12, respectively. The two complexes encode receptors that are very different and structurally unrelated. The *LRC* receptors have extracellular Ig-like domains that form the ligand-binding sites, whereas the extracellular domains of *NKC* receptors resemble the ligand-binding domains of C-type lectins. The ligands for many of these receptors are MHC class I molecules or molecules that in their structure and evolution are related to MHC class I molecules (7).

Initial studies of mice and humans showed that both species have a system of variable NK cell receptors that recognize polymorphic determinants of classical MHC class I molecules. Although the mouse Ly49 and human killer cell Ig–like receptor (KIR) systems are functionally similar, they are structurally unrelated. Ly49 receptors have extracellular lectin-like domains and are *NKC* encoded; KIR have extracellular Ig-like domains and are *LRC* encoded (8). That such structurally and genetically unrelated receptors perform analogous functions in two mammalian species is a striking example of convergent evolution and points to the dynamic selection pressures imposed on the immune and reproductive functions of NK cells.

The disparity of the variable NK cell receptors in humans and mice stimulated studies to determine what other placental mammals resemble humans in using KIR as variable NK receptors for MHC class I. In other simian primates, the *LRC* contains a family of *KIR* genes that corresponds to the human *KIR* gene family, but which also exhibits considerable species-specific character. For example, of 15 chimpanzee *KIR* genes, only three have strict orthologs among the 15 human *KIR* genes (9). In contrast with simian primates, the *LRC* of prosimian primates contains a single *KIR* gene and it is not functional (10). Either the prosimian primates have lost their families of *KIR* genes or they never had them, and the *KIR* systems we observed emerged and evolved specifically in the simian primates.

Study of nonprimate mammals shows that the *LRC* usually contains one *KIR* gene or has no *KIR* genes (11–15). A possible exception to this generalization emerged from studies of cDNA, which uncovered a diversity of expressed *KIR* in domestic cattle (16, 17). Phylogenetic comparison showed the cattle *KIR* formed two clades (18). One clade, represented by a single *KIR*, is most closely related to the family of expressed human *KIR*. The other, majority clade of cattle *KIR* is most closely related to the divergent, nonfunctional human *KIR3DX1* gene that is physically separated from the other *KIR* genes but still in the *LRC*. It lies at the center of the family of leukocyte Ig–like receptor (*LILR*) genes, which is adjacent to the *KIR* locus on the centromeric side (19).

These data are consistent with a model where duplication in the *LRC* of an ancestral *KIR*, occurring ∼135 million years ago before radiation of the placental mammals, produced the *KIR3DL* and *KIR3DX* daughter genes. Subsequently, successive duplication of the *KIR3DL* gene created the family of variable NK cell receptors of humans and other simian primates, whereas successive duplications of the *KIR3DX* gene created the diversity of functional KIR in cattle (18). Because nothing was known of cattle *KIR* genes, their genomic organization, and the cattle *LRC*, we determined the complete sequence of one domestic cattle *KIR* haplotype and the partial sequence of another. To assess the possible impact of domestication on the structure and diversity of cattle *KIR*, we compared the *KIR* genes in domestic cattle with their counterparts in the extinct aurochs ancestor of modern cattle.

## Materials and Methods

### Bacterial artificial chromosome library screening and clone sequencing

An existing Holstein–Friesian cattle bacterial artificial chromosome (BAC) library (20) was thawed and 5-μl aliquots from each well of the 290 ninety-six-well plates were pooled separately for each plate. After addition of an equal volume of water to this pool, the bacteria were lysed by a 10-min incubation at 96°C. This provided a template for standard PCR enabling each BAC plate to be rapidly screened for the genes of interest. Two pairs of primers were used to screen the library: one designed to amplify 3DL-lineage KIR (3DL_ex2_S1: 5′-CAKAGSATCTGGGCACAAG-3′, 3DL_ex3_AS3: 5′-GAATATGATGCCCTGGAGCTC-3′) and the other the 3DX-lineage KIR (3DX_ex3_S: 5′-GTCTCTCSCTGTGTTTTCCAG-3′, 3DX_ex4_AS: 5′-ATGACGATGTCCACAGGATCA-3′). In designing these primers, we used the published cattle KIR cDNA, as well as sequences of several KIR genes present in contigs from the cattle genome project (18). PCR was performed using GoTaq (Promega) with optimized cycling condition (95°C 1 min, [95°C 20 s, 62°C 20 s, 72°C 2.5 min] ×32, 72°C 5 min). A PCR amplicon of the correct size indicted that at least 1 of the 96 clones in the pool template contained a *KIR* gene. To identify the individual clones of interest, we then screened each well of that BAC plate individually using the same PCR primers and conditions.

This process identified six *KIR*^+^ BAC clones: 303D02, 369B10, 335H08, 095G08, 032G11, and 068F04. DNA was extracted from these clones using the Qiagen Large Construct Kit (Qiagen) according to the manufacturer’s guidelines. Plasmid DNA from 095G08, 335H08, 032G11, and 068F04 was sequenced at the Stanford Genome Technology Centre (Palo Alto, CA) using GS FLX titanium chemistry on a 454 instrument (Roche). BAC clone 303D02 was sequenced using the same chemistry except with 3-kb paired-end libraries at the Liverpool Centre for Genomic Research (Liverpool, U.K.).

### Fluorescence in situ hybridization of BAC clones

DNA samples from four of the *KIR* positive BAC clones, 303D02, 335H08, 095G08, and 032G11, as well as a BTA5 marker BAC (309A12) were labeled for fluorescence in situ hybridization by incorporation of one of five fluorescent-conjugated nucleotides using nick translation, as described previously (21, 22). Cattle chromosome preparations were generated using routine 72-h mitogenic stimulation of peripheral lymphocytes. After arrest at metaphase with a 30-min exposure to 50 ng/ml KaryoMAX (Life Technologies), conventional procedures of hypotonic treatment and fixation in 3:1 methanol/glacial acetic acid were used to harvest the metaphases from the lymphocyte culture. Chromosome preparations were dropped onto clean glass microscope slides, air-dried, dehydrated though an ethanol series, and stored at −80°C until required. Labeled BAC DNAs (50 ng each clone) were mixed with 10 μg sonicated cattle DNA before being hybridized to metaphase preparation for 18 h at 37°C. All hybridization and posthybridization steps were as described previously (21, 22). Chromosome preparations were counterstained with 80 ng/ml DAPI and mounted in antifade solution (Vectashield, Vector Laboratories). Multiplane images were acquired with a fluorescence microscope (Axioplan 2ie; Zeiss) equipped with suitable narrow-pass filter sets and a cooled CCD camera (CoolSnapHQ; Photometrics, Tuscon, AZ), both driven by dedicated software (SmartCapture 3; Digital Scientific, Cambridge, U.K.).

### De novo assembly of BAC clone sequences

BAC clones sequenced by 454 technology were screened for vector sequence (pBeloBAC11i) using SSAHA2 (23) and then assembled using the MIRA assembler version 3.4.0.1 (24) with genome, accurate, 454, and vector screen settings. Contigs from the initial MIRA assemblies were viewed and edited using the gap4 and gap5 programs from the Staden packages (25, 26) to join or break contigs, and also to design PCR primers to amplify regions of low coverage or span gaps between contigs. Contigs were broken when read coverage was less than four reads and overlap was <10 bp. Conversely, contigs were joined when read coverage was greater than four reads and overlap was >10 bp. PCR primers were designed within the regions between 100 and 500 bp from the end of each contig. Paired combinations of all contig end primers were then used for PCR using GoTaq (Promega) and the original BAC clone DNA as template (the PCR primers and conditions are available upon request). Amplicons were excised from agarose and the DNA extracted using a QIAquick gel extraction kit (Qiagen), and cloned using the pGEM-t vector systems (Promega). Plasmid DNA from at least three clones was extracted using a miniprep kit (Qiagen) and sequenced using vector end primers (M13F and M13R) with BigDye 3.1 chemistry (Life Sciences). Sanger sequences were edited for quality using pregap4 (26) before de novo assembly using the MIRA assembly with the raw 454 data. Initial assemblies indicated that these clones represented two distinct *KIR* haplotypes. BAC clones 095G08 and 335H08 overlapped and had identical sequences in the region of overlap, and to facilitate de novo assembly, the data sets for the two were combined. The sequences for clones 032G11 and 068F04 similarly overlapped and were analyzed in analogous fashion. Although these combined assemblies shared high-sequence identity, they were distinct from each other consistent with them representing two different haplotypes.

The sequence of BAC clone 303D02 did not overlap with that of any other BAC clone. To search for such overlapping sequence, we designed PCR primers at the ends of each contig. These primers were used in PCR with the same genomic DNA that was used to make the BAC library. For this PCR, primers corresponded to the ends of clones 303D02 (S 5′-CTGTTGGTGGGAATGCAAGC-3′) and 95G08 (AS 5′-GAAATCCACCTTGCTGTGCG-3′). Using GoTaq and the following conditions (95°C 5 min, followed by 32 cycles of 95°C 30 s, 54°C 30 s, 72°C 5 min), we amplified a 2-kb PCR product. Six clones of this amplicon were sequenced using the same system described earlier and incorporated into the 303D02 sequence database. This enabled the assembly of 1186-bp gap between the two completed assemblies. Subsequently, 454 sequences and the spanning Sanger sequences from 303D02 were merged into the same Staden database as 095G08 and 335H08 for manual assembly to create the complete *KIR* haplotype sequence. Ultimately, BAC clones 303D02, 095G08, and 335H08 were de novo assembled into haplotype 1. BAC clones 068F04 and 032G11 were de novo assembled into a second haplotype, haplotype 2. The nonoverlapping region of 032G11 with 068F04 failed to form one contiguous sequence and required finishing using a mapping approach with haplotype 1.

### KIR haplotype sequence verification and consensus generation

Four *KIR-*containing BAC clones (303D02, 335H08, 369B10, and 068F04) were sequenced using an Illumina HiSeq 2000 at ARK genomics (Roslin Institute, University of Edinburgh) using 500 bp insert libraries and 2 × 100 bp sequencing. Three of these (303D02, 369B10, and 068F04) had previously been 454 sequenced. The higher quality data obtained with the Illumina method (phred score > Q30) was used to correct homopolymer errors in the 454 sequences and to confirm the highly repetitive sequences in the *KIR* haplotype. Paired-end sequences were mapped to the de novo 454 assemblies using Burrows–Wheeler Aligner (BWA) with the “aln” algorithm and default options (27). Polymorphic sites were determined using samtools (28) and varscan2 (29) with a cutoff of 50.01% variant frequency and read coverage >5×. Positions that conflicted between different BAC clones or sequencing technologies were disregarded if the conflicting alignment had read coverage <8× (454) or 500× (Illumina). Consensus sequences were then generated using samtools mpileup output and a bespoke python script (available upon request) producing a majority consensus based on 51% base frequency and no ambiguity codes. The GenBank accession numbers for the cattle haplotypes assembled in this study are JX848345 (http://www.ncbi.nlm.nih.gov/nuccore/JX848345) for haplotype 1 and KM040762 (http://www.ncbi.nlm.nih.gov/nuccore/KM040762) for haplotype 2.

### Gene characterization

Consensus sequences of the finished BAC clone assemblies were examined for LRC gene exons using BLAT (30) searches using cattle *KIR* cDNA sequences as probes (*BotaKIR3DL3* EF197119, *BotaKIR3DL2* AF490402, *BotaKIR3DL2* NM_001098089, *BotaKIR2DS1* NM_001097567, *BotaKIR3DS1* NM_001008415, FCAR NM_001012685.1, NCR1 AF422181). To search for ITIM (canonical motif VxYxxL) and cattle ITAM (LLRL) motifs, which were not detected by BLAT searches, we visualized the assembled sequences in the Artemis genome browser (31). Repeat regions were annotated using the RepeatMasker Software (http://www.repeatmasker.org) and viewed alongside the LRC genes within Artemis. *KIR* gene sequences were extracted based on exon boundaries from the start of exon 1 to the end of the sequence encoding the cytoplasmic tail. Genes were aligned to known cattle cDNA sequences using MAFFT with default settings (32) and manually corrected using BioEdit (33) to produce predicted mRNA sequences for each gene.

### Phylogenetic tree construction

Sequences for all the cattle *KIR* genes identified within the BAC sequences were aligned with known cattle homologs/orthologs and *KIR* gene sequences from other mammalian species using MAFFT (32). Alignments were manually edited as necessary using BioEdit (33). Phylogenetic trees were constructed using MEGA 5 software (34) with either the Tamura-Nei or P-distance algorithm and bootstrapping of 1000 replicates. Inkscape software (http://inkscape.org) was used to annotate the trees. BAC *KIR* sequence exons were predicted after alignment with previously sequenced cDNA.

### Aurochs genome sequencing

All modern cattle, both taurine (*Bos taurus*) and zebu (*Bos indicus*), have descended from the extinct wild aurochs. These animals ranged widely across Africa, Asia, and Europe, and were domesticated on more than one occasion. The aurochs bone sample used to generate a complete genome sequence for this study has been previously described by us (35, 36). It consists of the proximal half of a humerus retrieved from Carsington Pasture Cave, Derbyshire, U.K. (http://capra.group.shef.ac.uk/1/carsing.html), which has been radiocarbon-dated to 6738 ± 68 calibrated years before present, securely dating CPC98 sample to the Mesolithic before the introduction of animal agriculture to the U.K. DNA extraction and purification from bone powder were performed in a dedicated ancient DNA laboratory as previously described (36).

### High-throughput sequencing of aurochs single-end libraries

Three Illumina single-read sequencing libraries (C1, C2, and C3) were prepared from independently sampled aurochs bone DNA extracts as described previously (36). For this study, aliquots of the C1 and C2 single-read libraries were subjected to additional agarose gel-based purification using a 4% agarose gel stained with 0.5 μg/ml ethidium bromide (Life Technologies) followed by extraction using a QIAquick gel extraction kit (Qiagen) to remove excess Illumina PCR adaptor dimer fragments. These C1 and C2 aliquots were then analyzed and quantified after gel purification on an Agilent Bioanalyzer using an Agilent DNA 7500 Labchip and sequenced across 16 flow cell lanes using an Illumina HiSeq 2000 sequencer. Sequencing was performed at the Beijing Genome Institute (Shenzhen, China) using 49-bp reads.

### Paired-end library preparation and sequencing

Three new independent DNA extracts (C4, C5, and C6) were generated from the CPC98 bone. The C4, C5, and C6 DNA extractions and subsequent paired-end sequencing libraries were prepared in a dedicated ancient DNA laboratory at the Department of Genetics, Trinity College (Dublin, Ireland). Powdered bone samples, weighing between 200 and 500 mg, were prepared using a modified procedure previously described by our group (35, 37) involving the addition of 200 μg/ml proteinase K to the DNA extraction buffer. DNA for each extract was eluted in 100 μl 1× Tris-EDTA buffer, divided into three separate 30 μl aliquots (labeled C4_1-3_, C5_1-3_ and C6_1-3_; giving a total of nine aliquots) and used to generate paired-end CPC98 sequencing libraries according to the Illumina Genome Analyzer (GA) Genomic DNA sample preparation kit protocol (Illumina, San Diego, CA). First, blunt end repair was performed on each DNA extract. For this, 30 μl aurochs DNA was included in a 100 μl final reaction mixture containing 1 × T4 DNA ligase buffer with 1 mM dATP (New England Biolabs [NEB], Ipswich, MA), 400 μM of each dNTP (Life Technologies, Paisley, U.K.), 15 U T4 DNA polymerase (NEB), 5 U DNA Polymerase I Large (Klenow) Fragment (NEB), and 50 U T4 polynucleotide kinase (NEB). Reaction mixtures were incubated at 20°C for 30 min, after which end-repaired DNA was purified using a QIAquick PCR Purification Kit (Qiagen, Crawley, U.K.) and eluted in 32 μl elution buffer according to the manufacturer’s instructions.

To facilitate Illumina GA adaptor ligation, we added a single “A” base to the 3′-ends of the blunt end–repaired aurochs DNA extracts. A total of 32 μl purified phosphorylated blunt end–repaired aurochs extract DNA was included in a final 50 μl reaction mixture containing 1× Klenow fragment buffer (NEB), 200 μM dATP (Life Technologies), and 15 U Klenow fragment with 3′-to-5′ exonuclease activity (NEB). Reactions were incubated at 37°C for 30 min, after which DNA was purified using a QIAquick MinElute Kit (Qiagen) and eluted in 19 μl elution buffer as per the manufacturer’s instructions.

Ligation reactions (in 50-μl vol) involved incubation of 19 μl phosphorylated blunt-ended aurochs DNA extracts, with a 3′-dATP overhang, with 1× DNA ligase buffer (NEB), 15 μM of each proprietary Illumina GA paired-end genomic adaptor (Illumina), and 10 U T4 DNA ligase (Life Technologies). Extracts were incubated at room temperature for 15 min, purified using QIAquick MinElute Kit (Qiagen), and eluted in 19 μl elution buffer according to the manufacturer’s instructions.

Individual paired-end Illumina GA libraries (*n* = 9) were generated via PCR amplification of the end-repaired adaptor-ligated DNA templates before sequencing. PCR amplifications (50 μl) comprised 19 μl blunt end–repaired linker-ligated aurochs DNA, 1 × Phusion High-Fidelity DNA polymerase buffer (NEB), 1 μl forward primer, 1 μl reverse primer (Illumina), 250 nM of each dNTP (Life Technologies) and 1 U Phusion High-Fidelity DNA polymerase (NEB). PCR amplification reactions consisted of an initial denaturation step of 98°C for 30 s, 12 cycles of 98°C for 10 s, 65°C for 30 s, and 72°C for 30 s, followed by a final extension step of 72°C for 5 min. Nontemplate controls were included with all PCR amplification reactions.

PCR products were visualized after electrophoresis on a 1.5% agarose gel stained with ethidium bromide (0.5 μg/ml; Life Technologies). Examination of the PCR products indicated the majority of the aurochs DNA inserts within the Illumina GA libraries were ∼40–60 bp in length. Individual libraries (*n* = 9) were subsequently pooled according to their initial extract number (C4_1–3_, C5_1–3_, and C6_1–3_) to form three final paired-end libraries labeled C4, C5, and C6, respectively. Pooled libraries were purified using a QIAquick PCR Purification Kit (Qiagen) and eluted in 30 μl elution buffer according to the manufacturer’s instructions. Purified libraries were quantified using a Qubit fluorometer (Life Technologies) and a Quant-iT dsDNA High-Sensitivity Assay Kit (Life Technologies). The final molar concentration of each of the three pooled libraries ranged between 0.25 and 0.31 μM (∼40.6–50.3 ng/μl).

For the pooled paired-end libraries C4, C5, and C6, cluster generation and sequencing was carried out on an Illumina cluster station and a GA II_X_ sequencer according to the manufacturer’s instructions. Libraries were sequenced using both single-read and paired-end sequencing. Three flow cells were used for single-end sequencing, using read lengths of 36, 42, and 70 bp. Three additional flow cells were used for paired-end sequencing, all using a read length of 42 bp. Analysis was performed using the standard Illumina GA pipeline. Intensity files generated by the IPAR server software were base called using Bustard (the Illumina base calling software). The first flow cell was processed using pipeline version 1.0. All subsequent flow cells were processed using pipeline version 1.3.

Raw genomic reads from aurochs DNA were aligned to the completed cattle *KIR-*containing region of the LRC using the BWA alignment software and default settings (27). Reads that aligned were extracted using the samtools package (28) and a custom python script (available upon request). The extracted reads were subsequently aligned to all *KIR* genes individually alongside a custom cattle genome build based on UMD 3.1 with all *KIR*-containing regions removed to ensure specificity to our assembled *KIR* genes.

Two approaches were taken to determine the presence or absence of individual *KIR* genes in the aurochs genome. Both approaches analyzed the variable positions between very similar *KIR* gene groups based on the two Holstein–Friesian haplotypes previously assembled. The aurochs genome sequence coverage at each position was also taken into account so that missing data were not mistakenly classified as the absence of a gene. In the first approach, variable positions between intragroup genes, including allelic variants from haplotype 1 and haplotype 2 where possible, were analyzed in the aurochs reads that uniquely mapped to that group of *KIR*. The position and number of variable positions that were consistent between the Holstein–Friesian and aurochs were calculated. The number of positions was then compared with the number of positions that were inconsistent or absent in the aligned aurochs reads. In the second approach, aurochs reads that mapped to haplotype 1 were filtered for unique map-ability by removing fragments without the “XT:A:U” tag, which denotes the read had mapped uniquely to that position. The percentage gene coverage was calculated using the bedtools package (38) and compared with positive control and simulated data sets. Simulated data were generated from the complete haplotype 1 sequence using a python script (available upon request) that produced artificial read fragments of 35 bp from both strands of DNA. Simulated read fragments were given a default quality score of phred 30, then aligned to the *KIR* haplotype using the same methods applied to the aurochs genome. This positive control coverage for each gene was calculated for positions with a depth >1000 reads. No positive control Illumina sequencing data were available for the BotaKIR3DL3*01 locus.

To examine individual polymorphic residues within each of the cattle *KIR* groups, we filtered aurochs genome reads that mapped to the cattle haplotypes 1 and 2 to remove any reads that mapped to any gene in another *KIR* group. This was achieved by using a bespoke python script (available upon request). Single nucleotide polymorphisms (SNPs) and indels were then called using the varscan2 software package (29), and the location and potential coding changes of the SNPs were determined using a bespoke python script (available upon request).

### Nomenclature

Cattle KIR, and the genes that encode them, were named according to the convention adopted for human KIR (39), but with addition of the prefix Bota to denote *Bos taurus.* The 3DL lineage KIRs are named according to the number of Ig-like domains (1, 2, or 3), the length of the cytoplasmic tail (S for the short tails of activating KIR or L for the long tails of inhibitory KIR), and they are then given a number in series. Thus, BotaKIR2DS1 and BotaKIR2DS2 are the first and second activating cattle KIR3DL having two Ig-like domains (D) and a short cytoplasmic tail. Names for the 3DX lineage KIRs are similarly assigned with the addition of *X* to the general descriptor. Thus, BotaKIR3DXL5 and BotaKIR3DXL6 are the fifth and sixth inhibitory KIR3DX having three Ig-like domains and a long cytoplasmic tail.

In implementing this nomenclature, some changes were required to the names previously assigned to nine cattle *KIR* cDNA. These assignments were made at a time when the differences between the 3DL and 3DX lineages were not yet appreciated (18). Where possible, previously named 3DX *BotaKIR* retain the same name with addition only of the *X* after the domain number (Supplemental Table 1). This was not the logical option for *BotaKIR3DL1P* and *BotaKIR3DL3*, which are allelic, and *BotaKIR3DL2*. Based on the position of these genes in the cattle *KIR* haplotype and their similarities to other *KIR*, these three *KIR3DX* have been renamed BotaKIR3DXL6*001N, BotaKIR3DXL6*002, and *BotaKIR3DXL4*, respectively (Supplemental Table 1).

## Results

### Cattle KIR genes map to the LRC near the telomeric end of chromosome 18

In most mammalian species, the *KIR* gene family, also referred to as the *KIR* locus, forms part of the *LRC* (8). In the current builds of the cattle genome (Btau 4.6.1 and UMD 3.1), both assembled using the same data from a Hereford cow, most *LRC* genes form an assembly that maps to a site near the telomeric end of chromosome 18 (BTA18). Among the genes in this assembly are genes that flank the *KIR* locus in other species: the *LILR* gene family on the centromeric side and the combination of *FCAR* and *NCR1* genes on the telomeric side. Notably absent from the assembly of cattle *LRC* genes are a majority of the nine known cattle KIR (16, 17, 40). Some *KIR-*like reads do map to the *LRC*, but others either map to the X chromosome (BTAX), the location of the two mouse *KIR* genes (41), or have not been mapped to any particular chromosome. To resolve these ambiguities and uncertainties, we undertook a targeted approach to characterize the cattle *KIR* genes and their location within the cattle genome.

**FIGURE 1.**
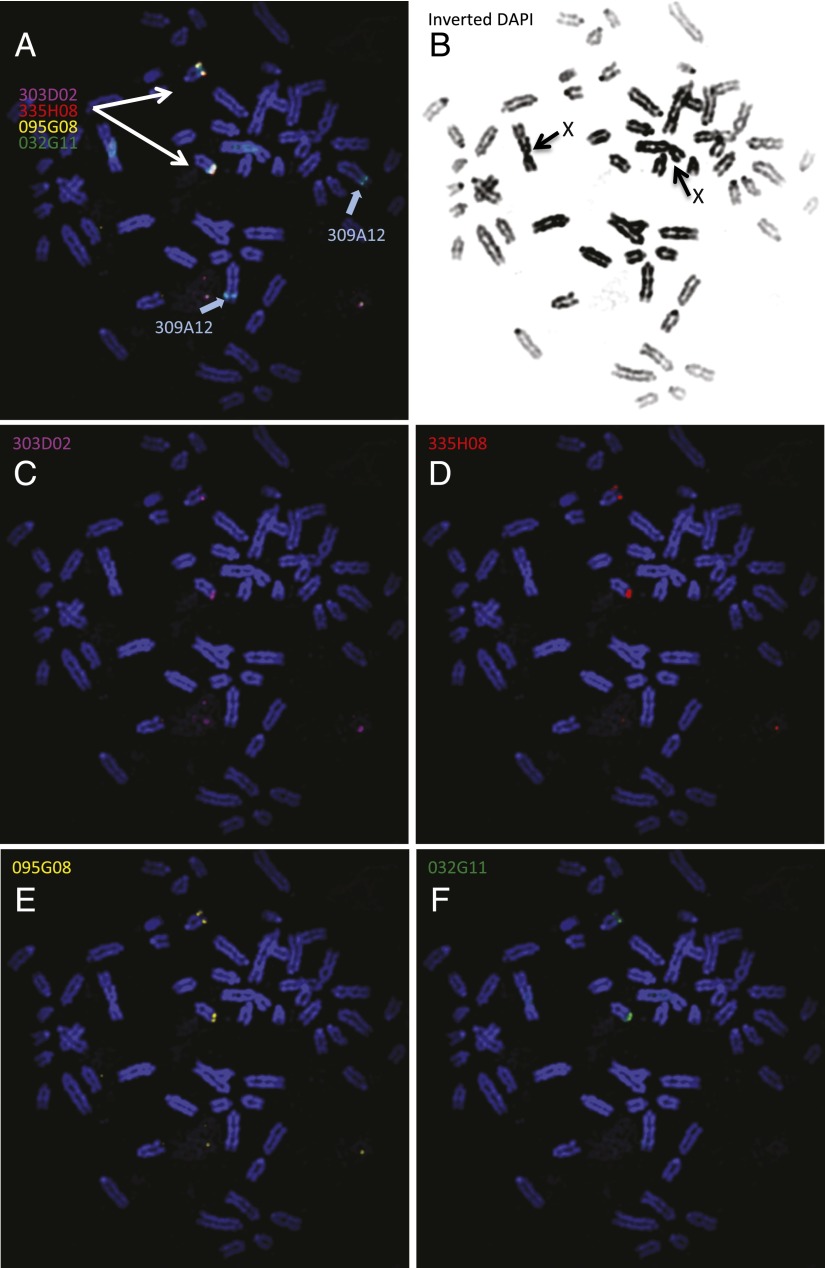
The cattle *KIR* cluster is exclusively located within the LRC on chromosome 18. (**A**) The *KIR*-containing BAC clones 303D02 (cherry), 335H08 (red), 095G08 (yellow), and 032G11 (green) all hybridize to the same site on one type of Holstein–Friesian chromosome. Because cattle are diploid, and both LRC and NKC are autosomal chromosomes, each BAC probe is seen to hybridize to two chromosomes of the same type. The control BAC clone 309A12 (blue) containing NKC genes maps to BTA5. (**B**) In contrast with the current genome assemblies, the *KIR* region does not map to BTAX. (**C**–**F**) Individual hybridization of the *KIR*-containing BAC clones. Original magnification ×1000.

A BAC library, made from the genomic DNA of one Holstein–Friesian bull (20), was screened by PCR amplification, using primers designed from the sequences of nine cattle *KIR* cDNA and the genomic *KIR*-like reads (16–18, 40). This screen identified six BAC clones that by sequencing were shown to contain a variety of cattle *KIR* genes, including ones corresponding to eight of the nine cDNAs. Four BAC clones, representing all the various *KIR* genes, were used as probes in separate hybridization experiments to a spread of Holstein–Friesian chromosomes. As a control, a BAC clone containing *NKC* genes was similarly analyzed. All four *KIR*-containing BAC clones hybridized to the same telomeric region of a single chromosome of approximately the same size as BTA18 (Fig. 1). In contrast, the *NKC*-containing BAC clone hybridized to a different chromosome of approximately the size expected of BTA5 (Fig. 1A), the known site of the NKC (42). None of the probes hybridized to BTAX (Fig. 1A). These results show that the *KIR*-gene containing inserts of the BAC clones all derive from a localized region near the telomere of a single chromosome. That the set of BAC clones includes the *LILR*, *FCAR*, and *NCR1* genes, as well as the cattle *KIR* genes, is consistent with the cattle *KIR* locus being part of the *LRC* near the telomeric end of BTA18. The precise organization of this part of the bovine *LRC* was then obtained from assembly of the complete sequences of the six *KIR*-containing BAC clones.

### Cattle KIR haplotypes contain multiple KIR3DX and KIR3DL genes

**FIGURE 2.**
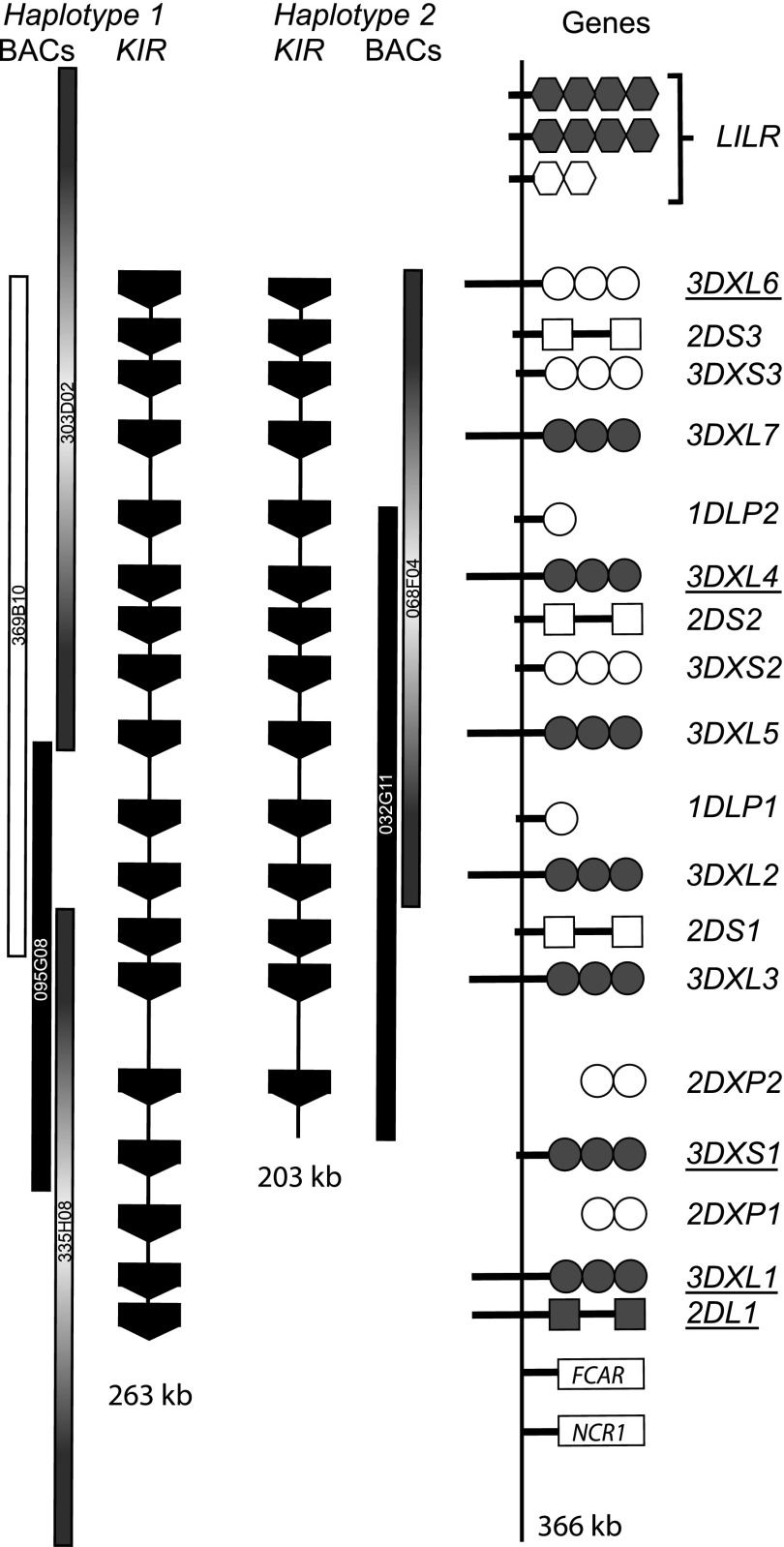
The cattle *KIR* complex is gene dense and contains multiple 3DL and 3DX-lineage *KIR*. Six BAC clones were sequenced using 454 pyrosequencing (black), Illumina 100 bp PE sequencing (white), or both (gradient), and assembled into two distinct haplotypes with identical gene content in the overlapping regions. The names of genes of which the cDNA were previously known are underlined. The length of the tail indicates if the gene encodes a short- or long-tailed receptor.

A contiguous 366-kb sequence was obtained from three overlapping BAC clones (303D02, 095G08, and 335H08). This assembly contains a complete *KIR* haplotype (*H1*) of 263 kb that is flanked on the centromeric side by three *LILR* genes and on the telomeric side by the *FCAR* and *NCR1* genes (Fig. 2). The haplotype consists of 18 *KIR* genes and gene fragments. The second *KIR* haplotype of the individual studied (*H2*) is partially covered by a 203-kb sequence obtained from two other overlapping BAC clones (032G11 and 068F04). This incomplete haplotype sequence corresponds to the centromeric two thirds of *H1* and includes 14 *KIR* genes and gene fragments; it is missing the first three exons of the most centromeric *KIR*, lacks the three *KIR* genes and one *KIR* gene fragment from the telomeric end of *H1*, and has no flanking non-*KIR* genes (Fig. 2). In the 203-kb region where both haplotype sequences are known, *H1* and *H2* have an identical gene organization but differ by 1008 SNPs. The sequences of the two haplotypes are clearly and significantly different, but exhibit no differences in gene content.

Eight of the 14 complete *KIR* genes of *H1* have open reading frames and are predicted to be expressed and functional. The other six complete *KIR* genes are probably nonfunctional because they have substitutions that disrupt the reading frame. Of the four *KIR* gene fragments, *KIR2DXP1* and *KIR2DXP2* comprise the exons encoding the D0 and D1 domains, whereas *KIR1DLP1* and *KIR1DLP2* comprise the exons encoding the D2, stem, and transmembrane domains (Fig. 2).

Eight of the nine cattle *KIR* cDNA sequences defined in previous studies (16, 17, 40) are products of the genes described in this article, although only one is identical, the allele of *BotaKIR3DXL6*. These comprise two alleles each for *BotaKIR2DL1*, *BotaKIR3DXL6*, and *BotaKIR3DXS1*, and one allele each for *BotaKIR3DXL1* and *BotaKIR3DXL4* (Supplemental Table 1). Thus, five of the eight functional *BotaKIR* are known to be transcribed. cDNA corresponding to *BotaKIR3DXL2*, *BotaKIR3DXL3*, *BotaKIR3DXL5*, and *BotaKIR3DXL7* have yet to be described. The one cattle *KIR* cDNA that is not represented in *H1* or *H2* is *BotaKIR2DXS1*. Thus, *BotaKIR2DXS1* could represent a *KIR* gene not present on *H1* and *H2*, or a divergent allele of one of the *H1* and *H2* genes.

### Evolution of the cattle KIR locus through duplication of genomic blocks

**FIGURE 3.**
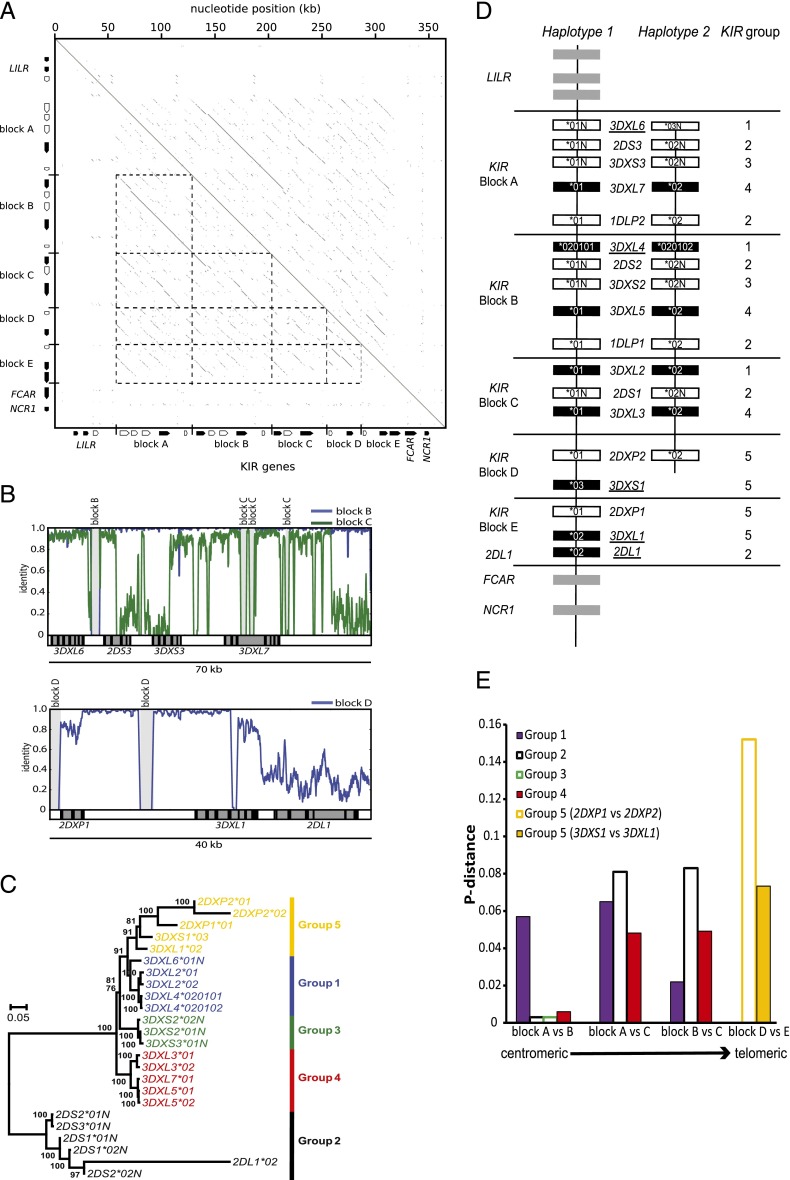
The cattle *KIR* gene cluster has expanded through multiple gene duplication events. (**A**) Pairwise analysis of the cattle *KIR* complex and flanking regions using dotter with a 250-bp sliding window (55). The family and position of each gene is indicated; filled are coding and open are noncoding. The blocks of high sequence identity within the *KIR* cluster are indicated, and the pairwise comparisons are boxed. (**B**) The sequence similarity between the related blocks A, B, and C within haplotype 1 using block A as the reference sequence (*upper panel*) and block D and E using block E as the reference (*lower panel*). A sliding window of 500 bp was analyzed, and the position of the genes within the reference sequence is shown below; gray rectangles show the area of the gene, and black lines are the exons within the gene. Vertical shaded gray columns represent unique sequence belonging to a block other than the reference sequence. (**C**) Phylogenetic analysis comparing the full-length gene sequences within both the cattle *KIR* haplotypes. Neighbor joining and maximum likelihood analysis gave trees with almost identical topology. Shown is a neighbor joining tree, rooted at the midpoint with the support for each node (expressed as a percentage) shown when >50%. (**D**) Scale diagram comparing the genes within each cattle *KIR* haplotype. Functional *KIR* genes are black, noncoding genes are open, and flanking genes are in gray. Genes encoding previously identified cDNA sequences are underlined, and the groups that each *KIR* gene belongs to are indicated. (**E**) Bar graph showing the P-distance between the *KIR* gene groups within each haplotype block. Mainly functional gene groups are indicated by filled bars and nonfunctional by open bars.

Dot-plot analysis of the *H1* sequence shows that the centromeric part of the *KIR* haplotype consists of two homologous genomic blocks of ∼66 kb in length, each containing three *KIRDX* genes, one *KIRDL* gene, and a *KIR1DLP* gene fragment (Fig. 3A). Sliding window analysis shows that these blocks, called *A* and *B*, have high sequence similarity throughout their length, consistent with the two blocks being the products of duplication of an ancestral ∼66-kb block (Fig. 3B). Consistent with this interpretation, phylogenetic analysis shows that each gene in block *B* is orthologous to the syntenic gene in block *A* (Fig. 3C). On the telomeric side of block *B* is a region of ∼51 kb, block *C*, which contains three genes (two *KIRDX* and one *KIR2DL*) that are orthologous to genes in blocks *B* and *A*, and are similarly arranged within the block. Sliding window analysis shows the *C* block has regions of high sequence similarity with blocks *B* and *A*, but is distinguished by a series of deletions involving three larger and several smaller regions (Fig. 3B).

Comparison of the *H1* and *H2* sequences shows that the two *A* block alleles differ by 177 substitutions and the two *B* block alleles differ by 191 nucleotide substitutions. The similarity of these numbers is consistent with the two blocks having a common origin and being the daughter products from the genomic duplication of an ancestral *A*/*B* block. The two *C* block alleles differ by 537 nucleotide substitutions, and although the mutation rate may not be consistent between all the blocks and alleles, this indicates that block *C* is likely to be significantly older than blocks *A* and *B*.

The genes present on both *H1* and *H2* are represented by a different allele on each haplotype (Fig. 3D). The number of SNPs between the exon sequences of all the block *A* and block *B* genes is 13 and 12, respectively. The difference between the exon sequences of the block *C* genes totals 40, despite possessing one less gene. With the exception of KIR3DXL4*020101 and KIR3DXL4*020102 within block *B*, the allele of each gene contains at least one polymorphism that produces a nonsynonymous substitution if translated. This again is consistent with block *C* being at least twice as old as blocks *A* and *B*, and reveals that the genes within each block are polymorphic.

The many differences that distinguish the *C* block from the *A* and *B* blocks indicate that there is no simple pathway connecting *C* with *A* and *B*, but that this evolution involved several duplications, deletions, and losses of intermediary forms. Between block *C* and *KIR2DL1* at the telomeric end of the locus are two homologous regions of 25 kb, each containing a *KIR2DXP* gene fragment and a functional *KIR3DX* gene (Fig. 3A, 3B). Block *D* encodes an activating receptor and block *E* an inhibitory receptor that share high identity, suggesting they could be functionally paired receptors with similar ligand specificity. These two regions were also likely formed by the duplication of a common ancestral region. Pairwise comparison between the homologous genes within each block along the *H1* haplotype shows that blocks *A* and *B* are very similar and that the distance between the nonfunctional genes is generally greater than the coding genes. The group 1 gene *KIR3DXL6* is, however, considerably more divergent than the other genes in block *A*, suggesting a more complicated evolutionary history.

The common ancestor of the placental mammals probably had one *KIR3DL* and one *KIR3DX* gene. That the full-length cattle *KIR* haplotype defined here has six *KIR3DL* and twelve *KIR3DX* genes and several gene fragments indicates that the *KIR3DL* and *KIR3DX* genes have been involved in at least three and five duplications, respectively. The duplication that yielded the *A* and *B* blocks is well defined, as is the duplication producing the telomeric region containing the paired *KIR3DXS1* and *KIR3DXL1* genes in blocks *D* and *E*. The nature of other duplications and deletions that contributed to the evolution of the cattle *KIR* haplotypes and their order are not well defined.

### Both ancient lineages of KIR genes were expanded during cattle evolution

**FIGURE 4.**
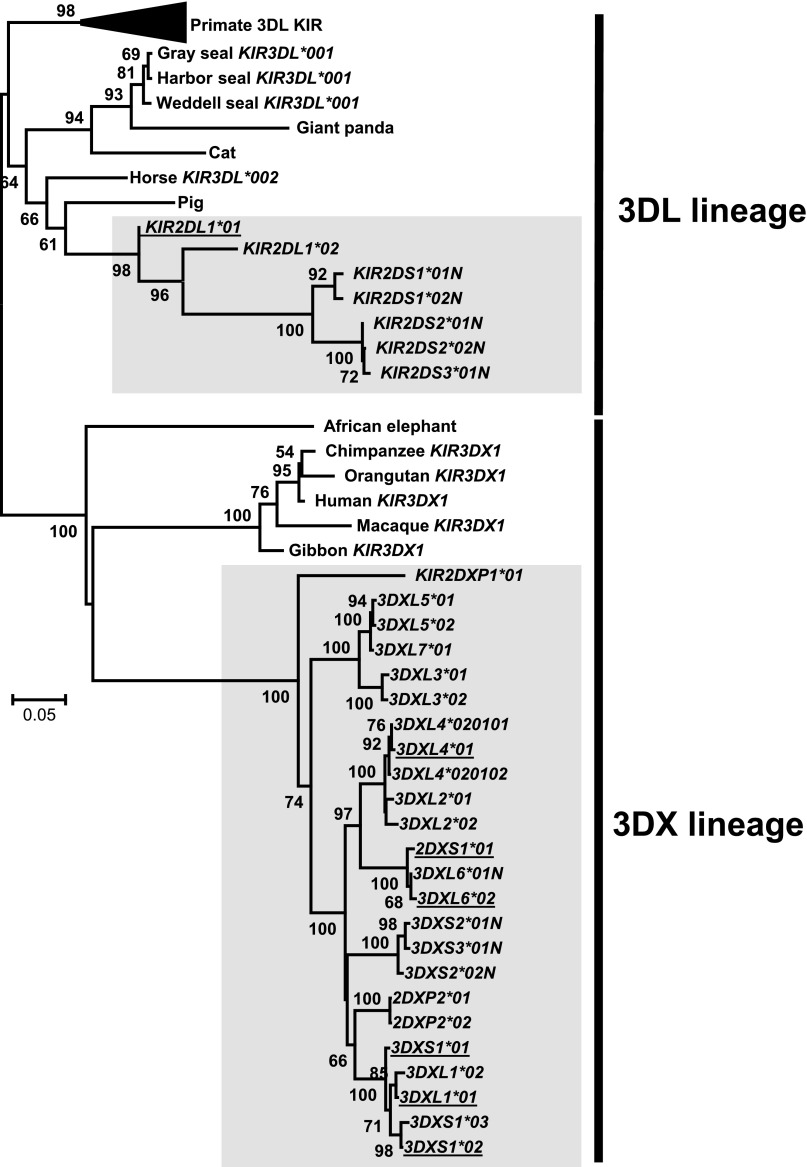
The cattle haplotype contains multiple 3DL and 3DX-lineage *KIR* genes with the same architecture as human *KIR*. Neighbor joining phylogenetic trees comparing all the known *KIR* exon 3, intron 3, and exon 4 sequences from cattle and selected genes from other mammals. Resolution between 3DX and 3DL-lineage *KIR* genes has previously been shown to deteriorate after the D1 domain, likely because of an ancient 3′ end interlineage recombination event (18), and all cattle *KIR* fit this model of evolution because we could not distinguish between lineages using the D2 domain.

The *KIR3DL* and *KIR3DX* lineages originated with duplication of their common ancestor ∼135 million years ago, before the diversification of modern mammals. In primates, the *KIR* gene family that is situated between *LILR* and *FCAR* comprises only *KIR3DL*, whereas the single *KIR3DX* gene is between the two halves of the *LILR* locus. Previous analysis of cDNA provided evidence for multiple cattle *KIR3DX*, but only for a single *KIR3DL* gene (16, 17). In this study, we demonstrate that the *H1 KIR* haplotype has six *KIR3DL* and twelve *KIR3DX* genes and gene fragments. However, *BotaKIR2DL1* is the only *KIR3DL* gene expressed, consistent with the earlier studies of cDNA. To our knowledge, cattle thus provide the first example of species in which both the *KIR3DL* and the *KIR3DX* genes have undergone expansion through duplication. This property, which distinguishes cattle from other species, is illustrated by phylogenetic analysis of D0-encoding exon 3, intron 3, and D1-encoding exon 4 (Fig. 4). In contrast with primates, the *KIR3DX* and *KIR3DL* genes have been adjacent throughout cattle evolution and have both been caught up in the duplications that expanded the *KIR* locus. That *KIR3DL* is maintained as a single functional gene, whereas functional *KIR3DX* have diversified, argues for positive selection on *KIR3DX* and negative selection on *KIR3DL*. By contrast, in primates, where a block of *LILR* genes separates the *KIR3DX* and *KIR3DL* genes, there is greater probability for duplications that involve *KIR3DL* without affecting *KIR3DX*.

### The major functional KIR in cattle are 3DX-lineage three Ig domain inhibitory genes

In contrast with the single functional *KIR3DL*, seven of the twelve 3DX-lineage *KIR* genes are predicted to encode functional proteins. Six of the seven functional 3DX-lineage genes encode receptors having three Ig-like domains and long cytoplasmic tails containing ITIM motifs. The one exception is the group 5 *KIR3DXS1*, which is predicted to have activating signaling potential and is a paired receptor with the inhibitory *KIR3DXL1* that formed during the duplication of blocks *D* and *E*. Therefore, the group 1, 4, and 5 genes encode the major functional KIR of cattle, which are defined as three Ig domain inhibitory receptors of the 3DX-lineage.

### Activating cattle KIR are short-lived and recurrently evolve

**FIGURE 5.**
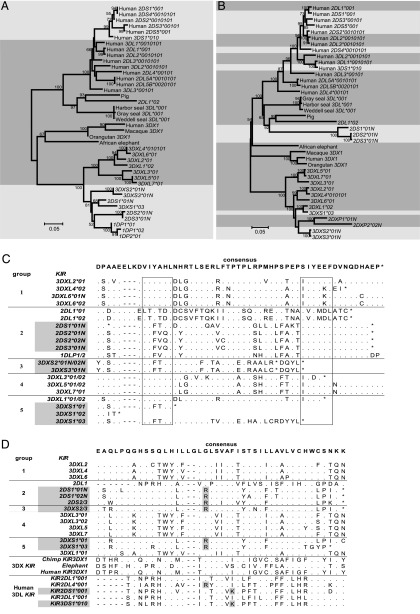
Activating cattle KIR evolved through recombination with a single short cytoplasmic tail. Neighbor-joining phylogenetic analysis comparing all the known cattle *KIR* cDNA and predicted cDNA sequences. The tree was rooted at the midpoint, and the support for each node (expressed as a percentage) is shown when >50%. Genes predicted to encode activating receptors are on a light gray background, genes predicted to encode inhibitory receptors are on a dark gray background. (**A**) Comparison of the coding sequence from the signal peptide to the D2 domain. (**B**) Comparison of the coding sequence and 3′ untranslated region from the transmembrane domain to the termination codon. (**D**) A comparison of the predicted transmembrane region of the cattle KIR with representative sequences from other species highlighting the charged residues present in short-tail receptors. The short-tailed receptors are shaded. (**C**) A comparison of the cytoplasmic region of the cattle KIR with the ITIM motifs boxed. The short-tailed receptors are shaded.

Of the six genes in the cattle *KIR* complex with the potential to encode an activating receptor, five are nonfunctional. Phylogenetic analysis of the region encompassing the sequence encoding the transmembrane domain through to the 3′ untranslated region shows a deep divergence between the genes encoding long-tailed and short-tailed receptors independently of the *KIR* lineage (Fig. 5A, 5B). The membrane-proximal ITIM motif of all the short-tailed cattle KIR has been disrupted at the same position and in the same way; the functionally important tyrosine within the canonical motif VxYxxL has been mutated to phenylalanine (Fig. 5C). This mutation is known to reduce binding to SHP-1 and SHP-2, and thereby attenuate inhibitory signaling (43).

Our analysis clearly shows that all the cytoplasmic tails of the short-tailed cattle KIR have a common origin, and that each different activating KIR has been formed by a recombination in which the long cytoplasmic tail of an inhibitory KIR has been replaced by this short tail. Because the deleterious mutations within the extracellular domains of various group 2 and 3 short-tailed *KIR* are identical (Supplemental Table 2), a single activating receptor within each of these groups must have acquired disabling mutations before the duplications of the gene blocks. As in cattle, the activating KIR of humans and the activating Ly49 receptors of mice have similarly evolved in a short-lived and recurrent fashion (44).

### Short-tail cattle KIR signal through Fcγ rather than DAP12

Activating receptors segregate into two major groups according to their associated adaptor protein. One group associates with Fcγ or CD3ζ through an arginine in a membrane-proximal position, and the other group associates either with DAP10 or DAP12 through a lysine residue within the transmembrane region (45). Unlike the activating primate KIR, which associate with DAP12, all past and present cattle short-tailed KIR are predicted to signal by association with a dimer of Fcγ (Fig. 5D). The *Fcγ* and *DAP12* genes are both present in the cattle genome and appear functional, which in the case of DAP12 has been demonstrated (46).

The *LILR* and *KIR* are related families of immunoreceptors that are encoded by adjacent *LRC* gene families in both primates and cattle (47, 48). Primate activating KIR associate with DAP12, whereas activating LILR associate with Fcγ. In contrast, activating cattle KIR and LILR are both predicted to associate with Fcγ, and phylogenetic analysis confirms that this is not due to recombination between the tails of these adjacent and related families (Supplemental Fig. 1). The homology between the genomic sequence encoding the transmembrane domain and flanking intron sequences of the short- and long-tailed KIR indicates that they evolved from the same ancestral sequence (data not shown). Therefore, despite the presence of both adaptor molecules, short-tailed KIR in primates and cattle have evolved independently and associate with alternative signaling molecules.

### Cattle KIR diversity

**FIGURE 6.**
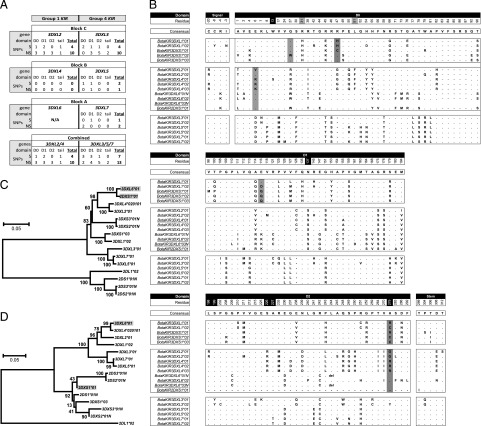
(**A**) A comparison between the predicted cDNA sequences from the functional genes present in blocks A, B, and C on both *KIR* haplotypes shows a concentration of nonsynonymous (NS) compared with synonymous (S) nucleotide substitutions mainly within the Ig domains. The region termed “tail” is from the stem to the stop codon. (**B**) Amino acid diversity between the diverse 3DX-lineage cattle *KIR*. All the previously known cDNA sequences (underlined) and the predicted cDNA sequences from this study were aligned, and residues that differ between or within the extracellular domains are displayed. Residues shaded in black are the equivalent residues in human KIR3DL1 that contact MHC class I (53), residue numbers shaded in gray are positively selected in human KIR3DL1 (54), and residues shaded in gray are predicted to be under positive selection after analysis using PAML (56). Identical residues to the consensus sequences are indicated with “.”; gaps are indicted with “-”; and “del” refers to a nucleotide deletion that disrupts the reading frame. (**C** and **D**) *BotaKIR2DXS1* is an allele of *BotaKIR3DXL6* with opposite signaling potential. Neighbor joining phylogenetic analysis with cDNA sequences of known cattle *KIR* with the predicted sequences from both the cattle haplotypes. Trees were rooted at the midpoint and node support is displayed when >50%. (C) Comparison of the coding sequence from the start codon to the end of the stem domain. (D) Comparison of the coding sequence from the transmembrane domain to the stop codon.

Variable haplotypic gene content and allelic variation diversify *KIR* in all higher primates and are maintained by balancing selection (49, 50). Therefore, we examined the polymorphism and associated signals of selection within the sequences encoding cattle *KIR.* For each functional *3DX*-lineage gene, the two haplotypes encoded different allotypic variants. Nucleotide polymorphisms are concentrated in exons encoding the Ig-like domains and dominated by nonsynonymous substitutions (Fig. 6A). The higher frequency of nonsynonymous substitutions than synonymous substitutions suggests that cattle *KIR* have diversified under pressure from natural selection. To address this question, we examined a data set of cDNA sequences, compiled from this study and previous reports (16, 17, 40), for the signature of positive selection. Likelihood ratio tests that consider codon variation in dN/dS (ω) gave strong evidence for positive selection in the Ig-like domains of the group 1 (*p* < 0.001) and the group 5 paired KIR (*p* < 0.001), and site analysis predicted that two (6 and 279) and four (27, 47, 116, and 279) positions had been subject to selection, respectively (*p* < 0.01; Fig. 6B). The common position under selection in both groups, 279, is the equivalent to position 278 in the D2 domain of human 3DL1 that is known to make contact with bound MHC class I ligand (51, 52).

*KIR3DXL6* is the most divergent of the group 1 *KIR* genes. Although the *H1* and *H2* haplotypes both have nonfunctional *KIR3DXL6* alleles that are noncoding, the previously reported KIR3DXL6*02 cDNA (40) appears functional and we have amplified several functional and nonfunctional *KIR3DXL6* alleles in studying a panel of cattle (unpublished data). In considering *KIRDXL6* polymorphism, it is important to note that the activating *KIR2DXS1* (16), the only cattle KIR cDNA that is not represented either on H1 or H2, has Ig-like domains that appear allelic to those of *KIR3DXL6* (Fig. 6C). Such similarity is not seen in the transmembrane domain and cytoplasmic tail where *KIR2DXS1* clades with the other activating KIR (Fig. 6D). These observations raise the possibility that activating *KIR2DXS1* and inhibitory *KIR3DXL6* segregate as alleles of the same gene. Precedent for this situation is the human *KIR3DL1/S*1 gene, which maintains a balance of activating KIR3DS1 and inhibitory KIR3DL1 allotypes (53). Recombinational analysis using RDP3 (54) indicates clearly (*p* < 0.01) that *KIR2DXS1* was formed by recombination between an allele of *KIR3DXL6* and one of the activating *KIR*. *KIR3DXL6* thus appears to be the most functionally diverse of the cattle *KIR*, having alternative forms on different haplotypes that encode activating receptors, inhibitory receptors, or are nonfunctional.

### The 16 KIR genes of domesticated Holstein–Friesian cattle are represented in the genome of an aurochs

Cattle, particularly the Holstein–Friesian breed, have been subject to intense artificial selection pressures during domestication. This history had the potential to reduce genetic variation, particularly for genes that evolve rapidly under natural selection. That the two Holstein–Friesian *KIR* haplotypes have similar structures could indicate loss of functional diversity during domestication, as could their accumulation of nonfunctional *KIR* genes. To assess the influence domestication had on cattle *KIR* evolution, we studied the *KIR* genes of an aurochs (*Bos primigenius*), the extinct common ancestor of modern domestic cattle. Aurochs DNA was extracted from a single adult humerus bone excavated from a cave in Derbyshire, U.K., and radiocarbon-dated to 6736 ± 68 y before present (36). Because this predates the influx of modern cattle from the Near East and the start of the Neolithic period in the U.K. by at least 800 y, this animal is unlikely to have been directly influenced by the human-imposed selection pressures involved in domestication.

**FIGURE 7.**
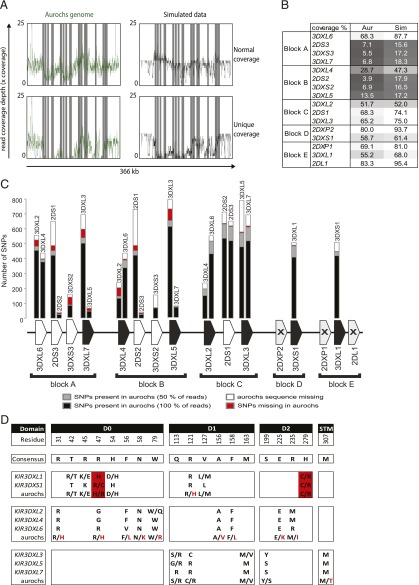
The undomesticated extinct ancestor of cattle contains the same *KIR* genes as a modern Holstein–Friesian bull. (**A**) Average read coverage depth of aligned short read sequences was calculated over a 300-bp sliding window. Normal coverage represents a short read dataset first filtered for reads that aligned to a version of the cattle genome assembly with the *KIR* reads removed, and then aligned to haplotype 1 using BWA. Unique coverage represents that same pool or reads filter for those that map equally well to more than one position on haplotype 1. Aurochs genome reads are shown in green. Simulated data (black) was artificially generated from the haplotype sequence then aligned to the haplotype using the same criteria. The location of the 16 *KIR* genes is shown by the vertical gray boxes. (**B**) Table of % coverage of read data for each gene using the uniquely mapping data. This was calculated based on the total length of the gene and number of positions covered by uniquely mapping reads. (**C**) The number of SNPs in the mapped aurochs genome reads that are identical to SNPs that define each gene in haplotypes 1 and 2 from the other *KIR* in that group. The reference gene being compared is indicated along the X axis in the same order and gene blocks as the sequenced haplotype. The genes being compared with the reference and the number of SNPs between each are labeled and numbered on the Y axis respectively. Genes marked with an X were precluded from this analysis as there was no significant identity to another gene. (**D**) Amino acid diversity with the 3DX-lineage group 1, 2, and 3 cattle *KIR* between the Holstein–Friesian and the aurochs. Residues shaded in red are predicted to be under positive selection in cattle using PAML (56). The consensus sequence was generated using the Holstein–Friesian data and all the nonsynonymous polymorphic sites in the aurochs are shown with the equivalent residues for each gene within that *KIR* group in the Holstein–Friesian. Residues in red are unique to the aurochs.

By applying multiple filtering processes to the short sequence reads of the aurochs genome sequence and mapping them on to the Holstein–Friesian *KIR* haplotype sequence, we identified the reads that locate exclusively to the *KIR* locus. Although these reads produced relatively even, but not complete, coverage of the entire *KIR* haplotype, ∼56% of the reads represent sequences that are present at more than one place in the haplotype (Fig. 7A). This was no surprise, given the short length of the ancient genome reads and high sequence similarity between the *KIR* genes. Filtering out the aurochs reads that map to more than one position on the haplotype significantly reduced coverage. To assess whether this reduction in coverage was due to the lack of *KIR* gene sequences in the aurochs genome reads or the highly identical and repetitive nature of the cattle haplotype, we applied the same filtering and mapping strategy to a simulated short read sequence data set based on the *H1* sequence (Fig. 7A). The pattern of mapping reads is very similar between the aurochs and the *H1*-simulated data with significantly reduced coverage at the 5′ end. This region contains the highly identical blocks *A* and *B*, indicating that the structure of the haplotype leads to a reduction in the amount of aurochs reads that map.

To assess which of the 16 Holstein–Friesian *KIR* genes is present in the aurochs genome, we identified the regions of sequence that are unique within each *KIR* gene. We then calculated the coverage of these unique regions by the aurochs genome reads. All the genes in the Holstein–Friesian haplotype had some uniquely mapping reads in the aurochs genome, but there was inevitably less coverage of the highly similar genes at the 5′ end of the haplotype, a trend again confirmed using the simulated data (Fig. 7B). We next selected for aurochs reads that mapped exclusively to one of the five groups of *KIR* genes. For each group, the group-specific reads were mapped onto each of the genes within that group. This enabled us to determine which of the polymorphisms that distinguish the members within a group are present in the aurochs data. Without allowing for any nucleotide ambiguity, SNPs unique for each gene within each group are present in the aurochs data (Fig. 7C).

This is evidence that every *KIR* gene within each block of the *KIR* haplotype is present in the aurochs genome, and reveals that the block duplication events at the *KIR* locus occurred at least 7000 y ago before the domestication of cattle. Furthermore, by varying the mapping stringency and examining the aurochs *KIR* reads that did not map to the Holstein–Friesian, we could find no evidence for the aurochs having any unique *KIR* genes, not present in domesticated cattle. The even coverage of Holstein–Friesian haplotypes by the aurochs data and the existence of no unique sequences raise the possibility that cattle *KIR* haplotypes are not as structurally diverse as human *KIR* haplotypes.

### Domestication has not altered the number of functional KIR genes

Although cattle domestication does not seem to have selected *KIR* haplotypes with gross changes in gene content, the Holstein–Friesian *KIR* haplotypes have several nonfunctional *KIR* genes that are disrupted by few nucleotide substitutions. We tested whether these same disrupting mutations were present in the aurochs genes. Although several SNPs were detected with high confidence, none of them disrupts the reading frame of a functional gene or restores the reading frame of the nonfunctional genes (data not shown). These predicted nonsynonymous mutations also reinforced the presence of each locus within the group 4 and group 5 genes, as gene-defining residues were present (Fig. 7D). However, within the group 1 genes, one residue at each position was unique to the aurochs likely being driven by the most diverse cattle *KIR* gene *BotaKIR3DXL6* (Fig. 7D).

## Discussion

This study establishes that the bovine *LRC* is on chromosome 18 and contains a *KIR* locus flanked by the *LILR* and *FCAR* genes that can account for all of the *KIR* genes predicted from previous cDNA sequence analyses (16, 17, 41). The sequence of one complete cattle *KIR* haplotype (*H1*) shows it contains 18 *KIR* genes. From the cattle *KIR* cDNA sequences and the genomic organization of the human *KIR3DL* and *KIR3DX* genes, we anticipated that an expanded family of cattle *KIR3DX* genes would accompany a single *KIR3DL* gene. This hypothesis proved incorrect. Twelve genes belong to the *KIR3DX* lineage and six to the *KIR3DL* lineage. Consistent with the cDNA analysis, the only *KIR3DL* gene expressed is *KIR2DL1*, whereas seven *KIR3DX* genes are predicted to be expressed with cDNA evidence for five. The cattle *KIR* locus is made up of a series of block duplications, each of which contains *KIR3DX* and *KIR3DL* genes, an arrangement that is different from that of the human *KIR* locus where the single *KIR3DX* is separated from the variable array of *KIR3DL* genes by several *LILR* genes.

A second cattle *KIR* haplotype (*H2*) includes 14 of the *KIR* genes, which have identical organization to their counterparts on *H1*. Furthermore, our analysis of *KIR* in the genome of an aurochs, the extinct progenitor of domestic cattle, is consistent with this individual having the same set of *KIR* genes as *H1* from a modern Holstein–Friesian individual. From this sampling, albeit a limited one, we obtained no evidence for cattle *KIR* haplotypes having gene-content variation, a common and defining characteristic of the *KIR* haplotypes in humans and other simian primates. Allelic polymorphism is a characteristic of cattle *KIR* genes, as is apparent from comparison of *H1* and *H2*.

Of the eight expressed cattle *KIR*, seven encode inhibitory receptors and one encodes an activating receptor. A similar bias toward inhibitory receptors is a feature of the mouse Ly49 and human KIR that recognize polymorphic determinants of MHC class I. In these species, the inhibitory function is associated with the education of NK cells to recognize damaged cells in which the expression of MHC class I has been perturbed by infection, malignancy, or other stress.

The properties of the cattle *KIR* genes that encode activating receptors are also remarkably similar to their human *KIR* and mouse *Ly49* counterparts. They combine divergent ligand-binding domains with very similar signaling domains (the transmembrane region and cytoplasmic tail) and they are short-lived compared with the inhibitory receptors. Emphasizing this latter point, five of the six cattle *KIR* encoding activating receptors were rendered nonfunctional by the acquisition of one or a few inactivating mutations, whereas only one *KIR* gene encoding an inhibitory receptor is inactivated, *KIR3DXL6*, which may be functional on other haplotypes. In summary, the results of this analysis of the structure and variability of the cattle *KIR* locus provide support for the hypothesis that cattle KIR are variable NK cell receptors for MHC class I.

However, a factor that must be considered in any cattle MHC class I/receptor system is the substantial diversity between *MHC class I* haplotypes. The coevolution of primate *KIR* with particular *MHC class I* genes and allele groups is well established. However, higher primates, especially humans, are unusual within Mammalia in having a relatively fixed number of highly polymorphic classical *MHC class I* genes. In cattle there are six classical *MHC class I* genes, with between one and three being present on any one haplotype. At its most extreme, two individual animals within a herd may not share any of their classical *MHC class I* genes. It remains to be seen just how diverse the cattle KLRC/D NK cell receptor system actually is, but the unique expansion of two receptor families in one species may have been influenced by the diversity of MHC class I.

It is striking that only one of the four 3DL-lineage *KIR* is functional, the single inhibitory gene. The three other 3DL *KIR* all arose by gene duplication from a single locus with the potential to encode an activating tail, after the original gene had been deactivated. Therefore, although there has been expansion of both lineages, the current evidence implies there has only ever been two functional 3DL-lineage KIR: one inhibitory and one activating. This may represent a functional constraint on *KIR* diversity that limits significant expansion to one lineage to maintain ligand interaction.

The preservation of gene content and coding sequence between an aurochs and a Holstein–Friesian bull indicates that domestication over the last 6700 y has not significantly altered *KIR* number or function. It is therefore reasonable to assume that despite altering selection processes, the *KIR* gene diversity that we observe in modern cattle evolved under natural selection. One striking outcome of this evolution is the dominance of 3DX-lineage three Ig domain inhibitory genes. It is clear from human and rodent research that the inhibitory genes are essential in educating NK cells to their host environment and licensing them to become effective components of the immune system. Compared with *KIR B* haplotypes, human *KIR A* haplotypes are dominated by inhibitory receptors and play an important role in NK cell education and resistance to infection. This suggests that cattle KIR are regulators of NK function during the immune response, and the allelic diversity we see in modern cattle likely affects differential immune responses to pathogens.

Interactions between variable NK cell receptors and polymorphic MHC class I ligands play crucial roles in immunity and reproduction; as a consequence, they are both rapidly evolving and highly species specific. Cattle and simian primates, including humans, are the only species known to have a diversity of *KIR* genes. This determination and analysis of the sequence of the bovine *KIR* gene family shows it is located in the bovine *LRC* in a comparable fashion with its human counterpart. The human and cattle KIR systems are independently evolved, but they exhibit properties in common that point to cattle KIR being variable NK cell receptors for MHC class I molecules. This work establishes the essential genetic foundation for studying the functional properties of bovine NK cells and their role in immunity and reproduction, as well as for population studies of KIR diversity and MHC class I diversity in herds and breeds of domestic cattle.

## Supplementary Material

Data Supplement
