# Supplementary material for: Definition of the Cattle Killer Cell Ig–like Receptor Gene Family: Comparison with Aurochs and Human Counterparts
Source: J Immunol. 2014 Nov 14;193(12):6016–30. doi: 10.4049/jimmunol.1401980 (PMC4258407; doi:10.4049/jimmunol.1401980)
Supplement: Data Supplement [file supp_193_12_6016__index.html]

Definition of the Cattle Killer Cell Ig–like Receptor Gene Family: Comparison with Aurochs and Human Counterparts — Data Supplement 

# Definition of the Cattle Killer Cell Ig–like Receptor Gene Family: Comparison with Aurochs and Human Counterparts

## Data Supplement

**Files in this Data Supplement:**

- Supplemental Material 1 (PDF)
