# Supplementary material for: Definition of the Cattle Killer Cell Ig–like Receptor Gene Family: Comparison with Aurochs and Human Counterparts
Source: J Immunol. 2014 Nov 14;193(12):6016–30. doi: 10.4049/jimmunol.1401980 (PMC4258407; doi:10.4049/jimmunol.1401980)
Supplement: Data Supplement [file 1401980_JI_1401980_Supplemental_Material_1.pdf]

Supplementary table 1

| Previous name | New name | KIR lineage | allele no | Breed | Reference | Accession | Description |
|---|---|---|---|---|---|---|---|
| *BotaKIR2DL1* | *BotaKIR2DL1* | 3DL | 01 | Unknown | 17 | AY075102.1 | Bos taurus NK receptor KIR2DL1 (KIR2DL1) mRNA, complete cds |
| *BotaKIR2DL1* | *BotaKIR2DL1* | 3DL | 01 | Holstein-Friesian | 16 | AF490399.1 | Bos taurus killer cell immunoglobulin-like receptor KIR2DL1 (KIR2DL1) mRNA, complete cds |
| *BotaKIR2DS1* | *BotaKIR2DXS1* | 3DX | 01 | Holstein-Friesian | 16 | AF490400.1 | Bos taurus killer cell immunoglobulin-like receptor KIR2DS1 (KIR2DS1) mRNA, partial cds |
| *BotaKIR3DL1* | *BotaKIR3DXL1* | 3DX | 01 | Holstein-Friesian | 16 | AF490402 | Bos taurus killer cell immunoglobulin-like receptor KIR3DL1 (KIR3DL1) mRNA, complete cds |
| *BotaKIR3DL2-001* | *BotaKIR3DXL4* | 3DX | 01 | Holstein-Friesian | 21 | EF197118 | Bos taurus KIR3DL2 (KIR3DL2) mRNA, KIR3DL2-001 allele, complete |
| *BotaKIR3DL1N* | *BotaKIR3DXL6* | 3DX | 01N | Unknown | 17 | AY075103.1 | Bos taurus NK receptor KIR3DL1-like protein pseudogene mRNA, partial sequence |
| *BotaKIR3DL3* | *BotaKIR3DXL6* | 3DX | 02 | Holstein-Friesian | 21 | EF197119 | Bos taurus KIR3DL3 (KIR3DL3) mRNA, KIR3DL3-001 allele, complete |
| *BotaKIR3DS1* | *BotaKIR3DXS1* | 3DX | 01 | Holstein-Friesian | 16 | AF490401 | Bos taurus killer cell immunoglobulin-like receptor KIR3DS1 (KIR3DS1) mRNA, complete cds |
| *BotaKIR3DS1-002* | *BotaKIR3DXS1* | 3DX | 02 | Holstein-Friesian | N/A | EF197120.1 | Bos taurus KIR3DS1 (KIR3DS1) mRNA, KIR3DS1-002 allele, complete cds |

The impact of the new Nomenclature for previously published cDNA sequences. Details of each cattle *KIR* gene cDNA submitted prior to publication of this paper are shown alongside the sequence's new designation and allele number.

Supplementary table 2

| Gene | Domain 0 | | Domain 1 | | | | Domain 2 | | | Transmenbrane | | Cytoplamic tail | | | |
|---|---|---|---|---|---|---|---|---|---|---|---|---|---|---|---|
| | mut. pos. (res.) | mut. pos. (res.) | mut. pos. (res.) | mut. pos. (res.) | mut. pos. (res.) | mut. pos. (res.) | mut. pos. (res.) | mut. pos. (res.) | mut. pos. (res.) | mut. pos. (res.) | mut. pos. (res.) | mut. pos. (res.) | mut. pos. (res.) | mut. pos. (res.) | mut. pos. (res.) |
| BotaKIR2DS1*01N | Stp 83 | Stp 93 | | | | | Stp 123 | Stp 127 | | Stp 197 | | | | | |
| BotaKIR2DS1*02N | mis. 81 | | | | | | | | | Stp 218 | | | | | |
| BotaKIR2DS2*01N | Stp 83 | | | | | | | Stp 136 | Stp 194 | Stp 213 | | | | | |
| BotaKIR2DS2*02N | Stp 83 | | | | | | | Stp 136 | Stp 194 | | | | | | |
| BotaKIR2DS2*02N | Stp 83 | | | | | | | Stp 136 | Stp 194 | Stp 213 | | | | | |
| BotaKIR2DS3*01N | Stp 83 | | | | | | | Stp 136 | Stp 194 | | | | | | |
| BotaKIR3DXL6*01N | | | | | | | mis. 229 | | | Stp 300 | Stp 326 | Stp 362 | Stp 372 | Stp 392 | Stp 409 |
| BotaKIR3DXS2*01N | Stp 11 | | Stp 111 | | Stp 148 | Stp 174 | Stp 220 | Stp 280 | | | | | | | |
| BotaKIR3DXS2*02N | Stp 11 | | Stp 111 | ins. 164 | Stp 149 | Stp 175 | Stp 221 | Stp 281 | | Stp 322 | | | | | |
| BotaKIR3DXS3*01N | Stp 11 | | Stp 111 | | Stp 148 | Stp 174 | Stp 220 | Stp 280 | | Stp 320 | | | | | |

Table of nullifying mutation positions within the cattle *KIR* genes. Each predicted null-allele or pseudoegene mRNA sequence was translated to elucidate premature stop codons or indels resulting in a reading frame shift. Positions shown are for the individual *KIR* genes and not the aligned positions.

Supplementary figure 1

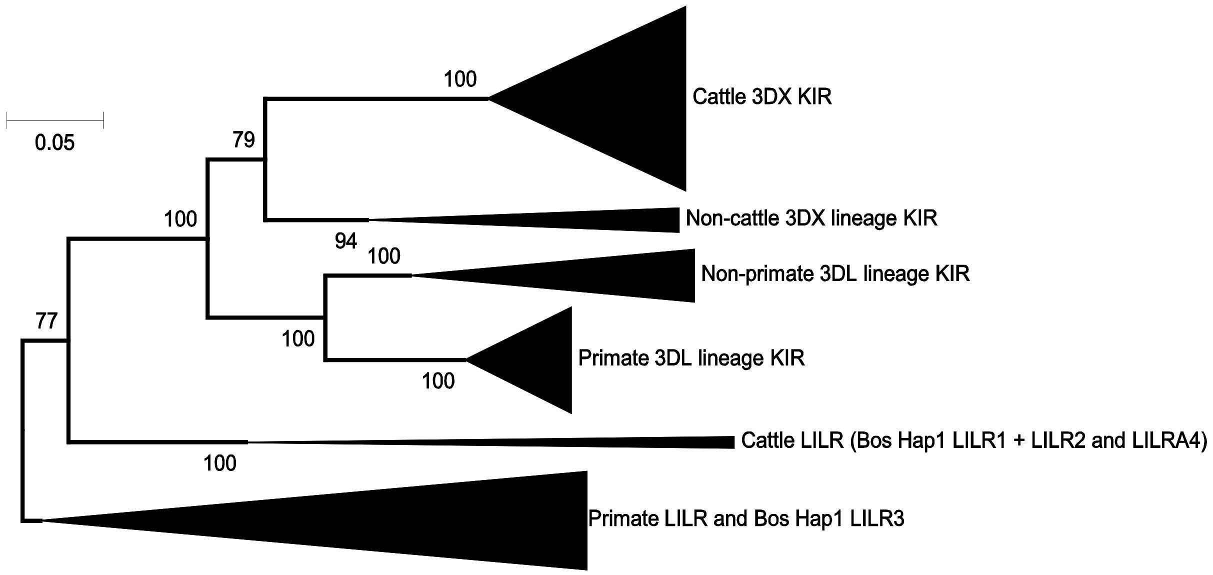


Supplementary figure 1.

Phylogenetic analysis of *LILR* and *KIR* genes using the transmembrane domain to cytoplasmic tail regions shows segregation of the two gene groups at the signalling end of the receptor. Groups of related genes have been collapsed to leave six clades in this tree, four are *KIR* and two are *LILR*. The P-distance tree with 100 bootstraps clearly shows a segregation of cattle *LILR* genes away from both primates and cattle *KIR* genes.
